# Impact of Habitat-Specific GPS Positional Error on Detection of Movement Scales by First-Passage Time Analysis

**DOI:** 10.1371/journal.pone.0048439

**Published:** 2012-11-07

**Authors:** David M. Williams, Amy Dechen Quinn, William F. Porter

**Affiliations:** Department of Environmental and Forest Biology, State University of New York College of Environmental Science and Forestry, Syracuse, New York, United States of America; Institut Pluridisciplinaire Hubert Curien, France

## Abstract

Advances in animal tracking technologies have reduced but not eliminated positional error. While aware of such inherent error, scientists often proceed with analyses that assume exact locations. The results of such analyses then represent one realization in a distribution of possible outcomes. Evaluating results within the context of that distribution can strengthen or weaken our confidence in conclusions drawn from the analysis in question. We evaluated the habitat-specific positional error of stationary GPS collars placed under a range of vegetation conditions that produced a gradient of canopy cover. We explored how variation of positional error in different vegetation cover types affects a researcher's ability to discern scales of movement in analyses of first-passage time for white-tailed deer (*Odocoileus virginianus*). We placed 11 GPS collars in 4 different vegetative canopy cover types classified as the proportion of cover above the collar (0–25%, 26–50%, 51–75%, and 76–100%). We simulated the effect of positional error on individual movement paths using cover-specific error distributions at each location. The different cover classes did not introduce any directional bias in positional observations (1 m≤mean≤6.51 m, 0.24≤*p*≤0.47), but the standard deviation of positional error of fixes increased significantly with increasing canopy cover class for the 0–25%, 26–50%, 51–75% classes (SD = 2.18 m, 3.07 m, and 4.61 m, respectively) and then leveled off in the 76–100% cover class (SD = 4.43 m). We then added cover-specific positional errors to individual deer movement paths and conducted first-passage time analyses on the noisy and original paths. First-passage time analyses were robust to habitat-specific error in a forest-agriculture landscape. For deer in a fragmented forest-agriculture environment, and species that move across similar geographic extents, we suggest that first-passage time analysis is robust with regard to positional errors.

## Introduction

Animal movement data are typically collected using very-high frequency (VHF) radio telemetry, and more recently, global positioning system (GPS) technology that record locations of animal positions in space and time. GPS technology has several advantages over VHF radio telemetry including finer spatial and temporal resolutions of location data and an ability to obtain positions remotely during harsh conditions and in hard-to-access locations. Moreover, the increased precision and accuracy of GPS tracking devices has allowed ecologists to evaluate animal behaviors and habitat use at increasingly finer scales. However, it is important to understand the limitations and biases of data acquired using GPS collars and the degree to which that error influences interpretations of data analyses.

For instance, as GPS collars attempt to acquire positional fixes at scheduled intervals (fix schedule), variations in behavior of the collared animal can affect the accuracy of the location [1], [2], [3], [4]. Similarly, collar model and manufacturer have been shown to influence accuracy and precision [4], [5], [6], [7]. The impacts of terrain on GPS collar positional error and bias are less clear, but appear to be greatest when elevations and slope gradients are large [8]. In landscapes with less rugged topography, positional accuracy is probably more affected by vegetative cover [9], [10], [11], and fix schedule [12], [13]. As a consequence of the positional errors arising from these factors, the set of locations recorded by a GPS collar represent one path among a distribution of paths that might have been recorded for that animal, rather than representing the actual path an animal travelled.

Movement analyses that do not account for uncertainty in GPS locations or the consequent distribution of possible movement paths, may lead to spurious results, insensitive analyses, or at best, limit the applicability of analytical conclusions. Jerde and Visscher [14] cautioned against modeling movement using step length and turn angle distributions based upon movements that were small relative to the standard deviation of positional error. Frair et al. [6] demonstrated that vegetation-specific fix-rate bias of GPS collars caused estimates of habitat selection parameters to fluctuate. Both groups stressed the need to run multiple simulations to stabilize those parameter estimates. Montgomery et al. [15], [16] found that telemetry positional error impacted the accuracy of resource use characterization. Moser and Garton [17] found that estimates of home range size using fixed kernel density estimators were unlikely to be influenced by positional error given adequate sample sizes. In simulations of movement, detection of movement scales was influenced by observation rate, missed fixes, positional error, and assumptions of movement responses to patches [18].

The first-passage time (FPT) analytical technique, which is increasingly used to quantify scales of movements, may be especially sensitive to error. FPT evaluates the variation of time spent within a specific area, or evaluation extent, along individual movement paths. Peaks in the variation of that passage time within a range of extents identify the scales at which each movement path is organized. These peaks have been interpreted as the landscape scales to which individuals are responding [19], [20]. Because small changes in a single positional observation could, at least hypothetically, determine whether an individual remains within a given evaluation extent or exits it, FPT may be sensitive to positional error. Additionally, the passage time of steps entering and exiting those evaluation extents is determined by dividing the length of those segments by the rate of travel for that step. Positional error will alter the distance traveled between fixes and thus affect inferences about the rate of travel for those path segments.

White-tailed deer (*Odocoileus virginianus*) that occupy the fragmented forest-agricultural landscape and rolling topography of central New York State are excellent organisms with which to test the influence of GPS location error on the detection of animal movement patterns. Deer in central NY typically utilize 2 distinct land-cover types: agriculture, which is open with little to no overhead vegetative cover, and forest, which ranges from heavy to light overhead vegetative cover [21]. GPS positional error is expected to be smaller in open areas than in heavy cover because of a reduced chance of radio signals being obstructed by dense canopy and a greater amount of sky available across which to locate satellites nearer the horizon [5], [7]. For similar reasons, GPS bias (failure to obtain a positional fix) is also expected when collars miss more fixes in heavy vegetative cover if they fail to receive signals from 3 or more satellites within the time interval designated to obtain a fix [8]. Any analyses of the movement of deer, particularly analyses that do not account for potentially distinct differences in observation error may be suspect.

The central question we address here is how robust are FPT analyses to positional error? Past research has investigated the influence of error on FPT analysis using simulations of movement in simulated landscapes [18], [22]. However, the problem becomes more challenging for a species like deer in a fragmented forest-agricultural landscape because they are moving between open (presumably low error) and forested (presumably higher error) habitat. The differences in error could have greater implications for identifying the scales at which their movements are organized. Thus, we sought to determine these influences using empirical data describing actual animal movement paths, where positional error is expected to differ along a path that passes through a heterogeneous landscape. Our objectives were to evaluate the influence of vegetation cover and fix schedule on positional error in GPS data and then explore how variation of positional error in different cover types would affect the ability to discern scales of movement in analyses of first-passage time.

## Methods

### Study area

**Figure 1:**
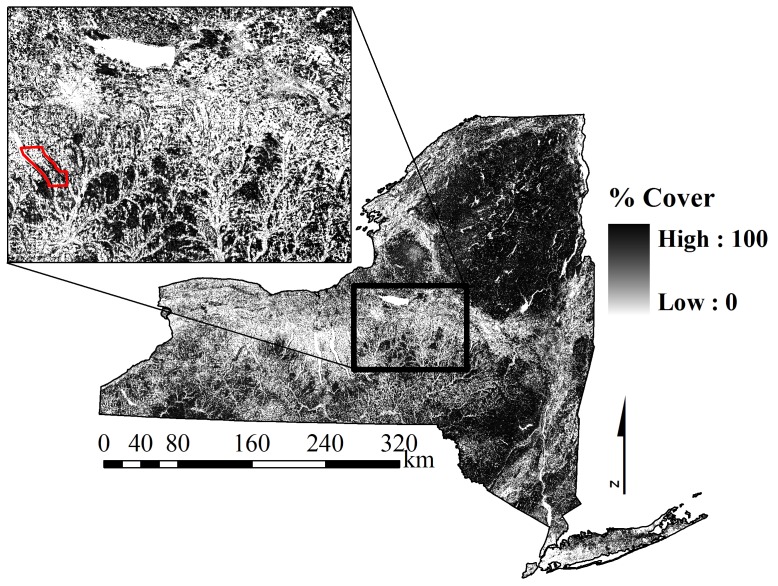
Percent canopy cover across New York State (Homer et al. 2004). Stationary collars were located within Spafford Township (outlined in red within the study area inset).

The study area included locations in Onondaga, Cortland, Madison, and Oneida Counties of central New York State (Fig. 1). Stationary collars were located within Spafford Township. Landcover was a mix of forest (44%) and agriculture (34%) with small communities (9% developed). Forests were dominated by hardwoods, notably sugar and red maple (*Acer saccharum* and *A. rubrum*), American beech (*Fagus grandifolia*), white ash (*Fraxinus americana*) and black cherry (*Prunus serotina*). Conifer plantations originating in the 1930's were composed of white, red and Scotch pine (*Pinus strobus*, *P. resinosa*, and *P. sylvestris*), and white and red spruce (*Picea glauca, P. rubens*). Agricultural crops were mostly related to dairy and include corn, winter wheat, oats, alfalfa, and soybeans. A rolling topography occurred throughout those portions of the study area in Onondaga, Cortland and Madison Counties; areas in Oneida County are located on a glacial lake plain. Average temperatures were −5.0°C during February and 20.6°C in July (1966–2006). Elevations range from 93 m to 652 m and the region lies to the south and east of Lake Ontario. The combination of the prevailing wind patterns and elevation affects precipitation. Average total annual precipitation was 97.3 cm/year (1966–2006). Winters are variable with heavy snow events and frequent thaws. Snowfall averaged 251 cm/year (1966–2006) and ranged from 241 cm/yr to 336 cm/yr during this study [23]. The deepest snowpack (74 cm) during our study occurred in Oneida County in February of 2007 [23]. Road density in the region was 1.85 km/km^2^; 1.5% of the landscape was >1.6 km from a road [24].

### Collar error

We placed 11 GPS collars (model GS2000, Advanced Telemetry Systems, Inc.) in 4 different vegetative canopy cover types classified as the proportion of cover above the collar (0–25%, 26–50%, 51–75%, and 76–100%). All collars collected data from 12 February 2008 to 11 March 2008. Collars were affixed to wooden stakes 1 m above ground level with the antenna oriented upwards and programmed with a primary schedule that attempted to acquire a position every 5 hr. Two of the collars in each cover class also attempted to acquire positions every 30 min for a 2 day period per 2 week interval. For the 0–25% cover class, we placed 3 collars in an agricultural field. For the 26–50% and 51–75% class, we placed 4 collars in mixed hardwood/coniferous forest (2 collars each). We placed the remaining 4 collars in a dense stand of conifers where vegetative cover was 76–100%. We used a handheld GPS Trimble unit (GeoXH) to record the true position of each collar as the mean of ≥25 fixes. We determined the percentage of missed positional fixes in each cover type for both the 5 hr schedule and 30 min schedule. We used the X and Y positional distance between each acquired location and the true position to evaluate potential positional bias and describe the standard deviation of an assumed symmetrical distribution representing the positional error for both axes of each fix-schedule and cover type combination. We used an F-test to compare the variances of the positional error distributions for each canopy cover class. We conducted model selection using Akaike's Information Criterion (AIC) to determine the best function relating the standard deviation of error for each collar to the percent canopy cover at each collar's location. We selected from null, linear, power, and logistic models to describe this relationship (Table 1). We conducted leave-one-out cross validation to evaluate the predictive accuracy of the best models. We identified the number of missed fixes to quantify any habitat (canopy cover) bias for acquisition of positional locations.

**Table 1:** Model comparisons for functional relationship between percent canopy cover and the standard deviation of positional error for stationary GPS collars, Spafford Township, NY.

Model		AIC	ΔAIC	ω*_i_*
Logistic	SD_canopy_ = *b*+(max-*b*)/(1+exp(a*(*m*-canopy)))	24.45	0	0.71
Linear	SD_canopy_ = *b*+*a* * canopy	27.01	2.56	0.20
Power	SD_canopy_ = *b*+*a* * (canopy*^p^*)	28.48	4.03	0.09
Constant	SD_canopy_ = *b*	45.18	20.73	0.00

### Movement data

We used GPS collar (model GS2000, Advanced Telemetry Systems, Inc.) data from 71 white-tailed deer (27 males and 44 females). Deer were captured during January-April 2006 and 2007 using modified Clover traps [25], rocket nets, and dart guns (State University of New York College of Environmental Science and Forestry Institutional Animal Care and Use Protocol no. 2005-1). Collars were programmed to take a GPS location every 5 hr. A secondary fix schedule acquired positions every 30 min for a 48-hr period every 2 weeks. GPS locations were stored on board the collars that were remotely detached from study animals and retrieved after approximately 1 year (mean = 254 days).

We simulated the effect of positional error on individual movement paths using the 5 hr fix schedule and cover- specific error distributions at each location. Estimated percent canopy cover at each observed deer location was extracted from the 2001 National Land Cover Tree Canopy Database for New York State [26] (Fig. 1). In our study area, percentage canopy cover as defined by this database was significantly spatially autocorrelated at lag distances ≤250 m and highly autocorrelated (*r*>0.7) for lag distances <100 m. Because percentage canopy cover is highly correlated at distances much greater than the average GPS location error, we used the percentage canopy cover of the 30 m cell underlying each observed GPS location. The best model describing the relationship between canopy cover and positional error was used to predict a site-specific error distribution at each observed GPS location. We randomly selected an X and Y distance from those distributions to relocate each observed point. We produced 500 iterations of the movement path for each deer using this habitat specific-positional error relationship.

### First-passage time analyses

We conducted FPT analyses of each deer movement path using the 5 hr fix schedule in the adehabitat package of the R [27], [28]. We calculated the passage time spent moving along the path within a circle of given radius that was centered on each GPS location along an individual movement path. Where the circle intersected the path between GPS locations we assumed constant rates of travel along the corresponding step and calculated time spent along the resulting path segment. We evaluated the passage time along each path using circles with radii ranging from 25 to 10,000 m at 25 m intervals. We identified the degree of aggregation of movements by the variation in passage time across all circles of a given size along the path. Because mean passage time and the corresponding variation around that mean are expected to increase as a function of increasing circle size we divided the variance in passage time by the area of the evaluation scale (circle size). For each deer, we identified peaks in the variance of passage time per unit area (varFPT/area) [19], [20]. Because FPT is an individual-based analysis, we identified common patterns in deer movement and randomly selected individuals, from a set of deer which exhibited those patterns, to identify the impact of positional error on the identification of peaks at different spatial scales. Once representative individuals were chosen, we repeated the FPT analyses at 10 m intervals across a smaller range where the peaks of interest occurred. Because positional error may be exacerbated by changes in animal behavior or seasonal changes to the canopy, we also evaluated the impact of greater positional error on FPT interpretation by increasing the predicted standard deviation of the observed error at each location by factors of 2, 5, and 10. For the randomly selected deer, we conducted 500 iterations of the movement paths as affected by these 3 additional error scenarios.

## Results

**Figure 2:**
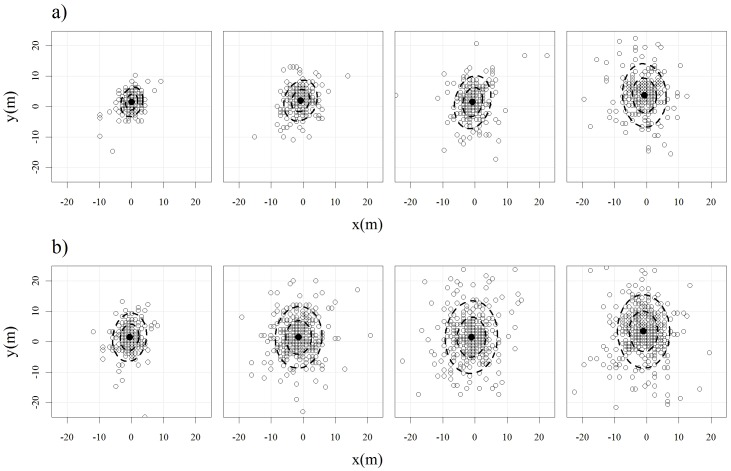
Positional error (m) of stationary collars placed in varying percentages of canopy cover in the Town of Spafford, New York from mid-February to mid-March 2008 for two different fix schedules: a) 5 hr and b) 30 min. Dashed lines represent the 50% and 90% normal ellipse contours of position densities. Percent canopy cover increases from left to right (0–25%, 26–50%, 51–75%, 76–100%).

We found that the standard deviation of positional error of fixes acquired on a 5 hr schedule increased significantly with increasing canopy cover class for the 0–25%, 26–50%, 51–75% classes (SD = 2.18 m, 3.07 m, and 4.61 m, respectively), but that there was no significant difference between the 51–75% and 76–100% cover classes (4.61 m and 4.43 m, F_306,259_ = 0.92, *p* = 0.51; Fig. 2). The relationship was similar for positions acquired on a 30 min schedule where the standard deviation of the positional error for the 0–25% (3.03 m), 26–50% (4.47 m), and 76–100% (5.29 m) cover class differed significantly (*p*<0.001 for all comparisons) (Fig. 2). For the 30-min fixes, the standard deviation of the positional error for the 51–75% canopy cover class (4.88 m) did not differ significantly from the 26–50% (F_387,387_ = 0.84, *p* = 0.085) or 76–100% classes (F_484,387_ = 1.17, *p* = 0.10).

**Figure 3:**
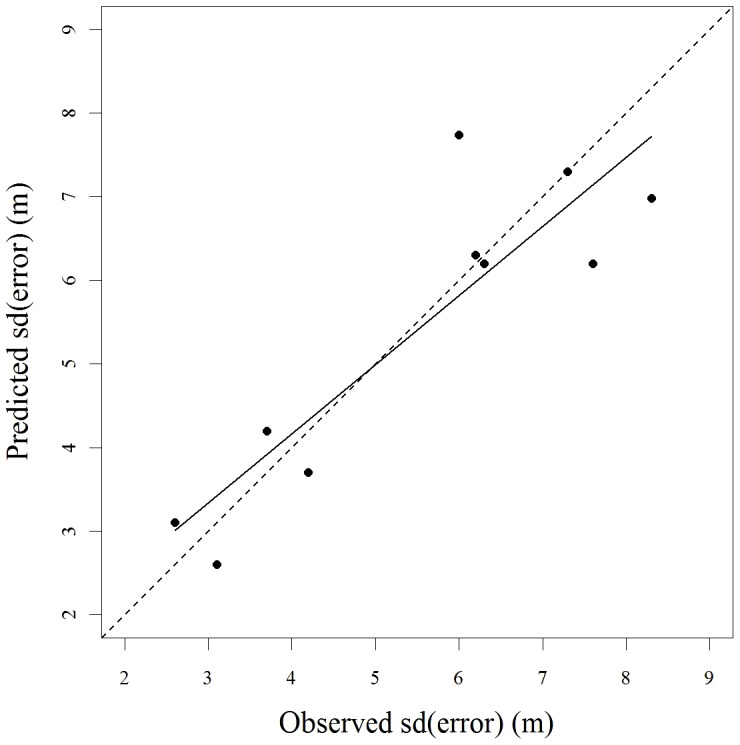
Regression of predicted versus observed standard deviation of error for leave-one-out cross-validation of the logistic model describing the relationship between percentage canopy cover and the standard deviation of the positional error of stationary GPS collars in central New York (*R^2^* = 0.79).

The relationship between the standard deviation of positional error for stationary collars and percentage canopy cover was best described by the logistic function. This model had 71% of the weight of evidence as the best model given the data and set of models (Table 1). Model validation indicated this model was a good predictor of the standard deviation of positional error. Observed and predicted positional error distributions were highly correlated (*r_s_* = 0.89, *p*<0.001) and their regression resulted in a slope near 1 (0.826, SE = 0.151)(Fig. 3).

We observed few missed positional fixes from the stationary collars in all canopy cover classes. We observed a 100% success rate for fixes acquired on a 30-min schedule regardless of canopy cover. Collars programmed to record position every 5 hr achieved 100% (*n* = 283), 99.62% (*n* = 261), 98.85% (*n* = 259), and 96.74% (*n* = 307) success rates for each cover class from least to greatest, respectively. We observed sequential missed fixes on only one occasion in the 76–100% cover class when two fixes were missed. Mean success rate for collars mounted on study animals was 86%.

### First-passage time

**Figure 4:**
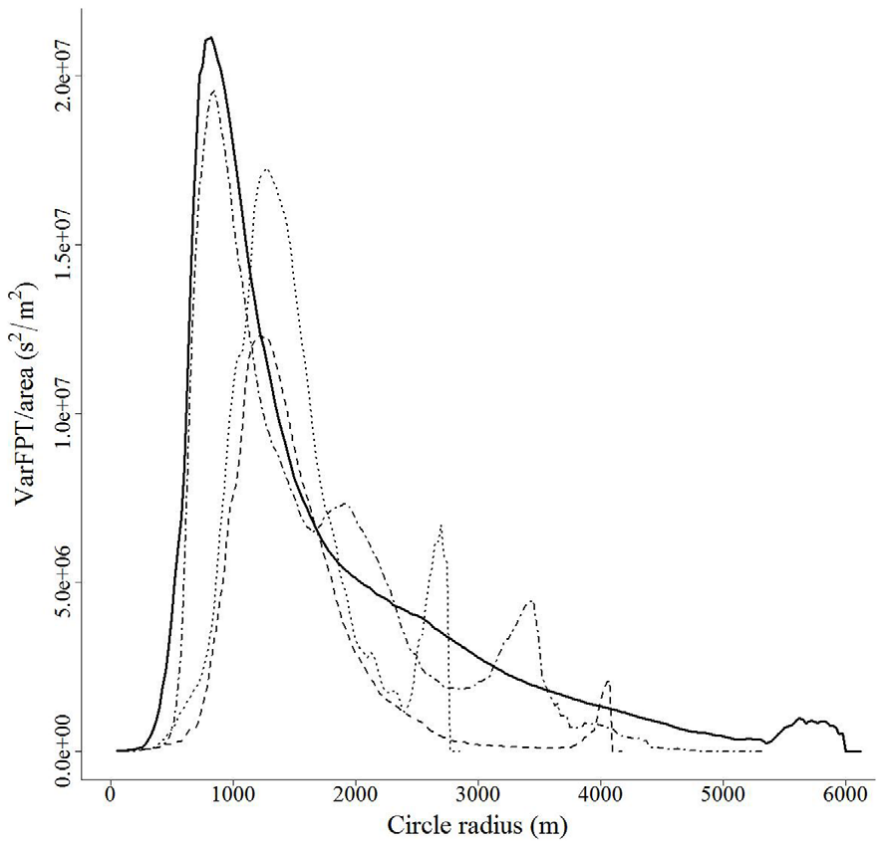
Plots of area adjusted variance in first-passage time (varFPT/area, s^2^/m^2^) as a function of the scale used to determine passage time along the observed movement path (radius of circle, m) for 4 white-tailed deer in central New York. Peaks in varFPT/area indicate the landscape scale(s) to which individuals are responding by altering time spent along their movement path.

**Figure 5:**
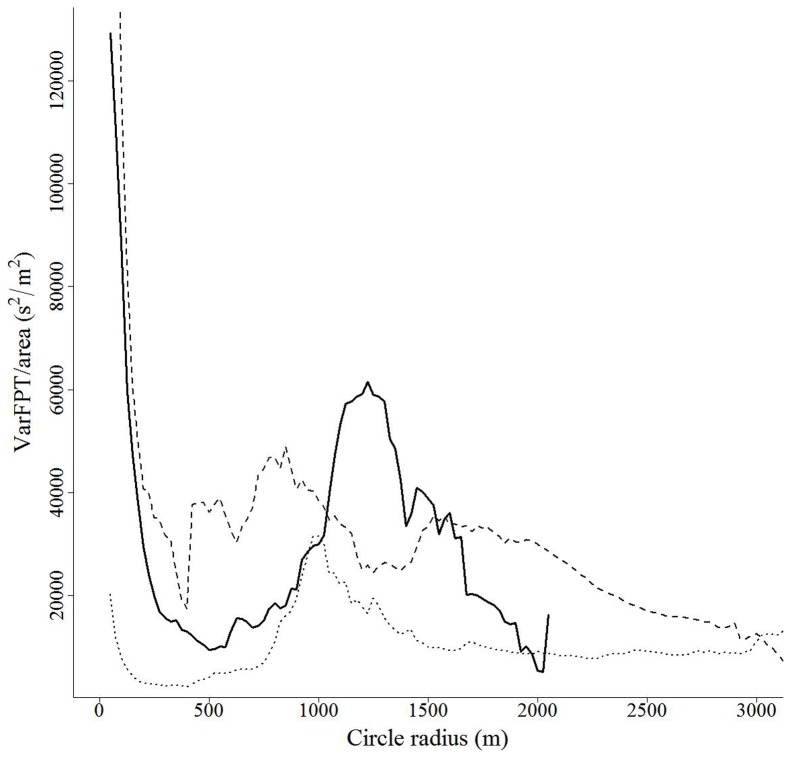
Plots of area adjusted variance in first-passage time (varFPT/area, s^2^/m^2^) as a function of the extent used to determine passage time along the observed movement path (radius of circle, m) for 3 white-tailed deer in central New York. Peaks in varFPT/area indicate the landscape scale(s) to which individuals are responding by altering time spent along their movement path.

White-tailed deer in central New York exhibited consistent scales of movement along their movement paths. FPT analysis for each individual across all seasons was dominated by a major peak in varFPT/area at scales (radii) from 575 m to 1,675 m (Fig. 4; [20]). We observed additional lower magnitude peaks at larger scales (3,000 m–6,000 m) for 32% (*n* = 20) of individual deer (Fig. 4). Twelve individuals (19%) exhibited high values of varFPT/area at the smallest scales evaluated that declined with increasing scale (25 m–150 m)(Fig. 5). No peaks in varFPT/area were observed for 8% (n = 5) of individuals.

### Impact of error on FPT peaks

**Figure 6:**
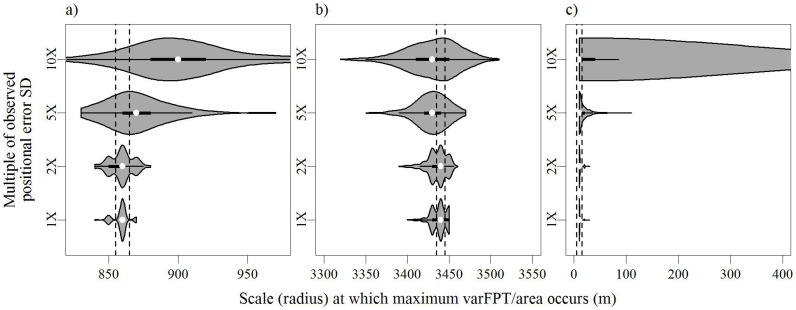
Violin-box plots displaying the impact of habitat-specific positional error on medium-(A), large-(B), and small-scale (C) peaks in varFPT/area for 500 simulated movement paths of white-tailed deer in central New York in 2006–2007. Dashed lines indicate the location of the peak in varFPT/area for the observed path (resolution = 10 m).

Scales of movement by deer as detected by first-passage time analyses were minimally influenced by habitat-specific error along the movement path. When we accounted for the observed positional error (1X) the peak consistently occurred within 10 m of the peak identified using the observed path. The varFPT/area as a function of scale along the path for deer #9, an adult female, was representative of analyses for many individuals exhibiting a dominant peak in the 425 m to 1,675 m range. For this individual, we found 73% of the iterations identified the detected scale as 860 m, and 99% of the iterations identified the peak in the range 850 m to 870 m (Fig. 6A). As we increased the standard deviation of the error (2X and 5X) incorporated into the movement path, on average the identified peaks remained consistent with the empirical peak, but the variance of the corresponding distributions increased. When we increased the observed habitat specific error by a factor of 10, the mean observed peak occurred at 900 m (SD = 40 m), a 40 m shift from the peak identified by the observed path (Fig. 6A). We found 95% of the dominant peak locations (centered on the mean) from the 10X error distributions ranged from 830 m to 970 m. This range included the location of the dominant peak in varFPT/area for the original path, although it is was observed rarely. Fewer than 6% of the observations of a normal distribution with a mean and standard deviation equivalent to that described by the 10X error peak locations are expected to fall between 855 m and 865 m, the location of dominant peak for the original path without error added.

Large-scale peaks in varFPT/area were also robust to potential habitat-specific positional error. The varFPT/area as a function of scale along the path for deer #101, another adult female, was representative of analyses for individuals exhibiting a large-scale peak in the 3,000 m to 6,000 m range (Fig. 6B). When we analyzed 500 paths resulting from incorporating the predicted positional error (1X) into the original movement path, we found 199 (39.8%) of the peaks were located at a scale identical to the one resulting from the original path (3440 m, Fig. 6B). The range 3430 m to 3450 m included 94.5% (*n* = 472) of the peak locations, and all of the peaks occurred between 3400 m and 3450 m. As we increased the amount of simulated error introduced to the observed path (2X, 5X, 10X), we found the mean scale at which the large-scale peak in varFPT/area occurred to differ by <10 m. The variation around that mean increased with increasing positional error. We observed standard deviations of 9.2, 11.3, 19.5, and 31.5 m for path iterations using the 1X, 2X, 5X, and 10X error functions, respectively (Fig. 6B). Twelve percent of the observations of a normal distribution with a mean and standard deviation equivalent to that described by the 10X error peak locations are expected to fall between 3,435 m and 3,445 m (the location of large-scale peak for the observed path). Fifty-five percent of the observations are expected to occur within a 50 m interval centered on 3440 m.

For all instances of peaks occurring at small scales we found that the peak identified using the observed path persisted despite the incorporation of positional error. The varFPT/area as a function of scale along the path for deer #68, an adult female, was representative of analyses for individuals exhibiting a small-scale peak at scales <100 m (Fig. 6C). For positional error simulations up to 5X the canopy-cover error function, nearly all iterations displayed peaks at scales <100 m (1X = 100%, 2X = 100%, 5X = 99.6%). When the canopy-cover error function was increased by a factor of 10 (representing a range of standard deviations from 25 m–70 m), 11.2% of the 500 iterations displayed no peak at scales <100 m.

## Discussion

While others have investigated the influence of various behavioral, landscape, and error changes upon FPT analyses using movement simulations and have evaluated the impact of positional error of ARGOS collars on FPT for small samples of albatross (Pinaud 2008), to our knowledge we are the first to examine the potential impacts of error on FPT analyses among a large number of GPS collared individuals in a natural landscape. We found mean positional error of GPS collars was <5 m across a full range of canopy cover conditions for deer in the fragmented forest-agricultural landscape of central New York. Few observed deer movements were smaller than the observed positional error of stationary collars (n = 794 of 71,095, 1.1%). This low positional error, when viewed in a context of the geographic extent of the movement we saw in deer, allowed for consistent estimates of the peaks in FPT analysis at all scales.

While mean positional error was low regardless of cover type, the standard deviation of the positional error of these collars was directly related to percentage canopy cover. Thus the spread of the potential locations around each observed location was greater as canopy cover increased. When we modeled that relationship, the best performing model was the logistic, suggesting that the distribution of positional error changes rapidly from low to high in corresponding canopy cover. Evaluation of the percentage canopy cover NLCD data for our study area reveals that when water is disregarded, 49% of the landscape is classified as having ≤25% overhead cover and 40% is classified ≥75% [26]. This dichotomy suggests that collars on deer moving through this landscape frequently function where canopy cover is associated with the extremes of their error distributions.

The positional errors of collars deployed on study organisms are potentially different from those identified by stationary collars. Unlike stationary collars, one cannot easily know the true location of each collared individual when GPS positions are recorded, and cannot identify the positional error of the recorded path. However, the difference in success rates of stationary versus deployed collars may inform whether we expect those to differ. Because we observed so few missed fixes among stationary collars, we did not evaluate the impact of missed fixes upon FPT analyses. It is important to note the difference between the success rate of stationary collars and those mounted on deer. We observed high success rates for acquiring positions among stationary collars across all canopy cover classes. For collars programmed on the 30-min fix schedule, this rate was 100% regardless of canopy cover; the success rate for the 5-hr schedule was also high ranging from 97% to 100%. However, the success rate among collars mounted on study animals (5-hr schedule) was lower (mean = 86%). This difference suggests that factors other than canopy cover are influencing the success rate of signal acquisition, and may imply that positional error of mounted collars may be greater than that observed among stationary collars.

Additional landscape characteristics may influence collar error and explain differences between the fix success of stationary and moving collars. Besides vegetation, topography has been found to influence GPS error. Topography impacts GPS error in canyons or mountainous terrain [4], [6], [12]. However, in less rugged terrain topography is seldom included in models describing GPS error. Dussault et al. [9] found no relationship between topographical metrics and GPS error. Their study area in eastern Canada had an elevation range of 250 to 1050 m. Our study area contained even less variation in elevation (range = 93 m–652 m, mean = 289 m, SD = 145.5 m) and thus we assumed differences in elevation did not impact collar error.

We suspect that the difference between the success rates of stationary and deployed collars is primarily due to being mounted on a moving organism where collar position varies with behavioral changes. Collar position (offset from vertical) has been found to negatively impact both position acquisition and location errors [2]. Researchers have found bedding behaviors in deer and moose to result in reduced fix success [29], [30]. Similarly, Graves and Waller [31] observed increased fix success with increased movement rates. More importantly they found that individual physical characteristics of bears explained most of the variation in acquired/missed GPS fixes. These studies demonstrate that collar performance is related to individual animal characteristics and behaviors. While these findings focus on missed fixes, it follows that positional error may also be related to these variables. Unfortunately it is difficult to know the true location of an individual at a specific time and thus, few have investigated the impact of behavior and motion on positional error. Cargnelutti et al. [3] evaluated GPS collar performance on a moving organism and found no difference in the positional error of stationary and moving collars, though stationary collars were mounted on tripods rather than study animals.

While we are unable to say whether the positional error is also greater for collars mounted on study organisms, it is likely given the increase in missed position acquisitions. This is the primary reason we evaluated the impact of positional error on FPT analyses using not only the positional error distribution observed among stationary collars, but also increasing multiples of the standard deviation (2, 5, and 10). The fact that for deer, FPT analyses were consistent even when positional error was assumed to be much greater than observed among stationary collars, indicates that even potentially unidentified sources of positional error are unlikely to influence detection of the scales at which deer movements are organized.

We found FPT analyses of movements for deer produced peaks in varFPT/area at 3 different landscape scales (small, intermediate, and large). We observed varFPT/area to be high at small scales (<50 m) and decline rapidly as scale increased for 12 individuals. For all other individuals we were unable to identify sufficient variation in varFPT/area to produce a peak at those scales. This may be a result of the temporal resolution of our data or variation among individuals. The 5-hr schedule for position acquisition may be too infrequent to identify variation in time spent along a path at scales <100 m. Similar peaks in varFPT/area were observed for individual elk (*Cervis elaphus*) in Alberta [19]. They interpreted these peaks as identifying the scale at which resting behaviors were occurring, with a caution to the potential impact of error on interpretation of the analyses at small scales. These small-scale peaks may also be artifacts of the analytical process [20]. We found these high values in varFPT/area occurring at small spatial scales to be consistent when we iteratively applied habitat-specific positional error to the observed movement path. Only upon incorporating error 10X greater than that observed among the stationary collars did the signal at small scales occasionally decline.

Using simulated movements in simulated landscapes, Pinaud [18] developed a log-linear relationship between the positional error and the smallest scales detectable by FPT analyses. The positional error of our GPS collars was much smaller than the Argos collars he was simulating, but if we extrapolate his findings they suggest that when the standard deviation of positional error is 8 m (the largest we observed among stationary collars in heavy canopy cover), the minimum area-restricted search one should be able to identify using FPT would have a radius of about 200 m. However, when small-scale peaks were present in our analyses, they persisted at scales <200 m despite simulated positional error.

The dominant scale, based on magnitude of varFPT/area, corresponded to the scale of seasonal space use. Dechen Quinn reported mean annual home range area of these deer as 1.9 km^2^ with variation among seasonal means: 1.1 km^2^ (spring-summer), 1.6 km^2^ (fall), and 1.7 km^2^ (winter) [21]. Deer in our study area typically occupied an area of the landscape during the spring/summer and fall seasons and migrated short distances to another area during winter [21]. When the positional error distributions of 5X and 10X were incorporated, we found both the variation and mean of the location of peaks in varFPT/area increased. This relationship suggests that as the cloud of potential locations for each observed location gets larger (increasing variation), the peak(s) in FPT analyses may shift to larger values. The shift we observed was <50 m, a distance that may have little meaning when identifying scales of seasonal space use for large species like deer.

Secondary peaks in varFPT/area occurred at larger scales corresponding to the distance separating seasonal space use or dispersals. Identification of these scales of movement and the interpretation of the corresponding landscape scale to which individuals are responding for these behaviors was robust to not only the positional error observed among stationary collars, but also multiples of that error. These peaks occurred at scales very large relative to the simulated positional error distributions. The range of peak locations for the greatest error simulations (Fig. 6B, 10X) spanned <200 m. This range suggests that even in situations where positional error is much greater than observed among stationary collars, FPT analyses can accurately identify organization of movement at large scales.

FPT analyses were robust to habitat-specific error in a forest-agriculture landscape where deer select for habitat representing the extremes of their positional error distributions and this suggests that our findings are applicable to other landscapes and species. Error associated with dichotomous use of open and closed habitat did not influence the spatial scale of detected peaks. Not only would we suggest that FPT analyses are expected to be robust in other forest-agriculture landscapes, but likely in other landscapes where positional error may be habitat- specific, and certainly in landscapes where the positional error distributions are similar across the landscape.

The ability to consistently resolve a peak in varFPT/area is undoubtedly influenced by the magnitude of the peak and the landscape scale at which the peak occurs. The strong signal for most deer in the intermediate range (425 m–1,675 m) makes it very difficult for small changes in position along the path to reduce variability in time spent along the path. Additionally the intermediate and large-scale peaks occurred at landscape scales 2 orders of magnitude larger than the positional error of the collars. This is important because we can assume that FPT analyses for other GPS collared species that exhibit area-restricted searches at scales >400 m are robust. We limited our positional error simulations to 10X that observed by stationary collars. Thus, if positional error is expected to be very large, >100 m, one may consider evaluating the impact of error on FPT analyses.
